# Adaptive language model training for molecular design

**DOI:** 10.1186/s13321-023-00719-7

**Published:** 2023-06-08

**Authors:** Andrew E. Blanchard, Debsindhu Bhowmik, Zachary Fox, John Gounley, Jens Glaser, Belinda S. Akpa, Stephan Irle

**Affiliations:** 1 Computational Sciences and Engineering Division, Oak Ridge National Laboratory, Oak Ridge, TN 37831 USA; 2 National Center for Computational Sciences, Oak Ridge National Laboratory, Oak Ridge, TN 37831 USA; 3 Biosciences Division, Oak Ridge National Laboratory, Oak Ridge, TN 37831 USA; 4 Chemical & Biomolecular Engineering, University of Tennessee, Knoxville, TN 37996 USA

**Keywords:** Masked language model, Drug discovery, Genetic algorithm

## Abstract

The vast size of chemical space necessitates computational approaches to automate and accelerate the design of molecular sequences to guide experimental efforts for drug discovery. Genetic algorithms provide a useful framework to incrementally generate molecules by applying mutations to known chemical structures. Recently, masked language models have been applied to automate the mutation process by leveraging large compound libraries to learn commonly occurring chemical sequences (i.e., using tokenization) and predict rearrangements (i.e., using mask prediction). Here, we consider how language models can be adapted to improve molecule generation for different optimization tasks. We use two different generation strategies for comparison, fixed and adaptive. The fixed strategy uses a pre-trained model to generate mutations; the adaptive strategy trains the language model on each new generation of molecules selected for target properties during optimization. Our results show that the adaptive strategy allows the language model to more closely fit the distribution of molecules in the population. Therefore, for enhanced fitness optimization, we suggest the use of the fixed strategy during an initial phase followed by the use of the adaptive strategy. We demonstrate the impact of adaptive training by searching for molecules that optimize both heuristic metrics, drug-likeness and synthesizability, as well as predicted protein binding affinity from a surrogate model. Our results show that the adaptive strategy provides a significant improvement in fitness optimization compared to the fixed pre-trained model, empowering the application of language models to molecular design tasks.

## Introduction

The goal of rational drug design is to identify molecules with specified properties associated with therapeutic value. Emerging infectious diseases (e.g. SARS-CoV-2 and the associated pandemic) highlight the need for rational design to accelerate the discovery of drugs in response to novel protein targets [1, 2]. Computer aided drug discovery (CADD) provides a set of tools to shorten the time and cost of searching chemical space for new applications [2–7]. In addition to the development of biophysical models and simulations traditionally associated with CADD [5–7], much recent work has focused on using methods from machine learning (ML) and artificial intelligence (AI) for molecular design [4, 5, 7–9].

The use of ML models in drug design has been enabled by the availability of large compound libraries [10] and experimental datasets [11, 12] along with computational libraries for cheminformatics [13]. Within a design application, models generally serve one of two possibly overlapping roles, molecule generation and molecule scoring. Generative models, such as variational autoencoders [8, 14] and generative adversarial networks [15, 16], are capable of sampling new molecules from chemical space based off a training set. Scoring models, on the other hand, take a molecule as input and generate a prediction for a given property (e.g. protein binding affinity). Through iterations of generation and scoring, searches over chemical space can be performed to optimize a given property. The iterative process for optimization is commonly referred to as a genetic algorithm [17].

Genetic algorithms provide a useful strategy for the design of molecular sequences for drug discovery applications. To use a genetic algorithm, a representation for a chemical sequence must be chosen along with a mutation operator to generate new sequences. The mutation operator is then used to explore chemical space and selection is performed according to a pre-defined fitness objective. Previous studies have used genetic algorithms successfully for a range of drug discovery applications [18–22]. Furthermore, benchmark studies have shown that genetic algorithms can achieve state-of-the-art results for molecule generation, comparing favorably to recent machine learning techniques [19, 21].

Despite the success of genetic algorithms, the need to define an appropriate representation for a chemical sequence and a mutation operator poses a challenge. Previous studies have often utilized a simple representation by enumerating individual atoms and bonds within a molecule [18, 19, 22]. For mutation, hand-crafted rules, such as add an atom, delete an atom, or create a ring, have been proposed and used for large scale exploration of chemical space [18]. Additional studies have used data mining techniques to discover commonly occurring multi-atom fragments and used custom mutation operators to rearrange the specified fragments [20, 22–25]. However, specifying fixed rules for rearrangements limits the ability to adapt the optimization procedure to a given task. Ideally, the mutation operator can be automatically inferred from the data, reducing the need for intuition and generalizing the genetic algorithm approach to new molecular design tasks.

A related approach to molecule generation utilizes recurrent neural network (RNN) based architectures such as the Long Short-Term Memory (LSTM). More generally, statistical language-based models utilize different structural representations (e.g., molecular fingerprints) for generation and optimization based architectures. For example, Segler et al. [26] had showed how a LSTM based models can be used for transfer learning as they are fine-tuned on smaller population of molecules to achieve activity towards certain biological target and thus be used to generate novel set of molecules with desired activities. Along that direction, Arés-Pous et al. [27] have carried out an extensive study on different RNN based models (such as LSTM and Gated recurrent unit or GRU) using different Simplified Molecular Input Line Entry System (SMILES) representations like canonical, randomized and DeepSMILES versions. These different experiments designs are then tested on various sizes of molecule populations ranging from 10k to 1 million. In another recent RNN based work [28] on two different string representations namely SMILES and SELF-referencing Embedded Strings (SELFIES) demonstrated that RNN-based language models can deliver powerful generative capabilities while learning complex chemical rules of the molecular representations better than graph-based models. This observation is then further extended by the works of Awale et al. [29] when they trained LSTM based generative models on different datasets including full size drug molecules along with fragments and performed transfer learning to demonstrate that fragments-based training is as capable as training on full size molecules in producing efficient drug analogs. In related work on biogenic compounds Zheng et al. [30] developed a quasi-biogenic molecule generator (QBMG) with GRU RNN to generate quasi-biogenic compounds, libraries including stereochemistry and a de novo approach to produce focused libraries influenced by certain scaffolds. On the other hand, recent proposed methods based on conditional generative adversarial networks [31] or GAN, offers an alternative strategy to take advantage of all information stored in compound-induced gene expression data to generate active-like molecules. As their method requires no explicit activity or target annotation information during training process, this can be used as a target-independent generalized approach. But algorithm wise these types of models are very different than bidirectional transformers-based models. Transformer based large language models (LLM) are different than RNN or LSTM type language models. These transformer-based molecule generators in recent times demonstrate how effective these LLMs could be in designing novel molecules for different purposes as required. Bidirectional Encoder Representations from Transformers (BERT) [32] based LLMs showed advantages while tested on established benchmark models and datasets for downstream tasks and gCT [33] (i.e., generative chemical Transformer) showed improved or at-least on-par performance. Similarly generative pre-training (GPT) [34] models delivers comparable performance in generating novel, valid and unique molecules when tested on benchmark datasets with other models.

The present work i.e., a novel strategy about how to generate a new population of molecules resembling initial highly optimized molecules by adapting the original optimized properties while restricting from generating a generic broader population distribution of new molecules, is a direct improvement over using fixed pre-trained model as demonstrated. Under-the-hood our implementation is based on Transformer architecture specially to be mentioned as Bidirectional Encoder Representations from Transformers (BERT) [32]. This particular type of architecture has shown proven advantage when used on established benchmark datasets such as GuacaMol [21] for targeted benchmark tasks such as virtual screening and QSAR applications by positively impacting subsequent downstream tasks, augmenting the constancy of learnt molecular representation and improved performance over present dataset [32]. In related work using transformers model on chemical designing, analogous architecture namely gCT [33] (i.e., generative chemical Transformer) also able to successfully generate valid new molecules that satisfy various required target properties while showing either improved (or at-par in some cases) compared to other benchmark reference models (such as MOSES models [35]). Also, on using related large language models (LLM) based architecture such as using generative pre-training (GPT) [34] models we see results and performance that are comparable to previously implemented machine learning algorithms to task like designing valid, novel, and unique molecules when compared with MOSES [35] benchmark models and datasets.

Inspired by the advances in natural language processing (NLP) [36], recent studies have shown how to automate both the choice of representation for chemical structure and the mutation operator [2, 37]. Starting with a text-based representation for molecules, SMILES [38], the process of tokenization is used to determine commonly occurring subsequences [39, 40]. The subsequences are stored as a vocabulary and are used to map a given molecule sequence into a list of token IDs. Each token ID may correspond to multiple characters (i.e., atoms and bonds) in a given molecule. Once a tokenization scheme is defined, the molecule data can be used to train a masked language model. In the training for such a model, tokens are randomly masked and the loss is determined by how well the model reproduces the original sequence when predicting the masked tokens [36].

**Fig. 1:**
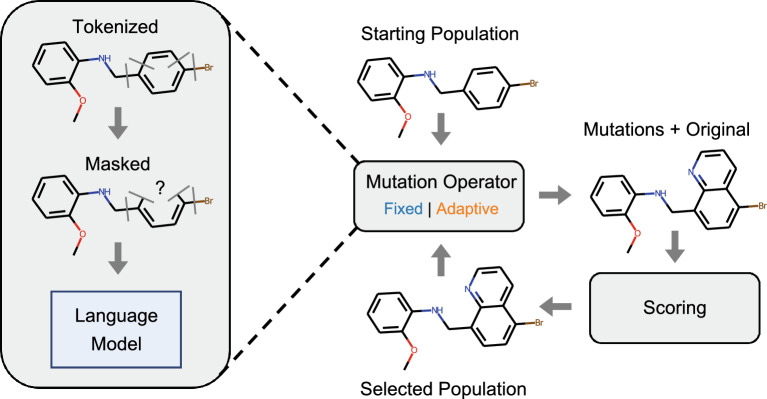
Strategy for molecule optimization using a language model. An initial population of molecules is used as input. The language model then generates mutations using predictions for randomly placed masks. Molecules are ranked according to a specified score and top performers are selected for another round of mutations. Two approaches for the language model are investigated, fixed and adaptive. For the fixed approach, the language model is pre-trained on a large molecule dataset and it does not change during the optimization process. For the adaptive approach, the language model is trained on the selected population, which itself changes during the optimization process

Without the need for labels, unsupervised training of masked language models can be performed on large compound libraries (e.g. Enamine *REAL* database) [10]. For a given mask, a trained model will rank possible ways to complete the molecular sequence based on the vocabulary. Therefore, sampling from the top mask predictions provides an automated mutation operator for a genetic algorithm [37]. Therefore, in contrast to manually defining rules for mutations, masked language models provide an automated solution for discovering both useful molecular representations (i.e., through tokenization) and mutations (i.e., through mask prediction) as shown in Fig. 1.

Although the use of a fixed pre-trained masked language model provides a useful improvement over manually defined rules, the challenge to adapt molecule generation for different optimization tasks remains. For example, the dataset used for model pre-training may have certain biases that limit structural rearrangements useful for a new task. In order to overcome this difficulty, we here propose a novel way to use language models within genetic algorithm optimization. Specifically, we continue to train the masked language model on populations selected for a specified fitness objective. By continued training on the selected population, we hypothesized that the language model would adapt to new regions of chemical space useful for optimization.

In order to test our hypothesis, we implemented two approaches for comparison - fixed and adaptive. In the fixed approach, a pre-trained language model was used to generate new molecules. In the adaptive approach, the pre-trained language model is used as a starting point and further trained using mask prediction on a specified population. Continued training is performed after each iteration of the genetic algorithm to produce a new population of molecules. Our results show that the adaptive approach produces data that more closely mimics the genetic algorithm population. For optimization, the adaptive approach leads to increases in fitness for tasks using both heuristic metrics and a ML surrogate model. Therefore, by introducing the adaptive approach for automating mutations we broaden the capabilities of genetic algorithm optimization for molecular design.

## Methods

### Genetic algorithm

In this work, we focused on the molecule generation capabilities of a masked language model for fitness optimization. The source code for this work can be found at https://code.ornl.gov/candle/mlmol in the adaptive-lm directory. As described in previous work [37], a masked language model can be used as an automated mutation operator within a genetic algorithm. Figure 1 shows the major components for optimization. An initial population of molecules, in the form of SMILES strings are used as input to the masked language model. Portions of a given SMILES string are then randomly masked and the language model is used to predict mutations to the original molecule. The generated molecules are then scored and selection is performed based on the specified fitness to generate an optimized population. The process of mutation and selection can be repeated for a specified number of iterations.

For the language model acting as the mutation operator, we considered two different training strategies, fixed and adaptive. In both cases, we started by pre-training a masked language model on a dataset with billions of molecules (for further details on the dataset, see Methods Section - Molecule Data). For the fixed strategy, weights of the pre-trained model were frozen, and the model was used only for inference (i.e., mask prediction) as part of the genetic algorithm. For the adaptive strategy, however, model training based on mask prediction was performed for one epoch during each generation, with the current population of molecules used as the training data. The language model, therefore, adapted to the patterns found in the current population of the genetic algorithm before generating mutations.

To distinguish between the optimization performance of the fixed and adaptive strategies, we utilized a relatively simple genetic algorithm with a $(\mu + 5\mu )$ survivor selection scheme. Random uniform sampling with replacement was used to select $\mu$ parents from the population, and only mutation was used to generate new molecules, similar to our previous work [37]. A population size ($\mu$) of $10^5$ was used for all reported genetic algorithm simulations. Mutations were generated by taking the top 5 predictions from the masked language model for a given set of masks. Validity and uniqueness of the generated molecules were determined using rdkit [13] to convert SMILES strings into canonical form. Only unique molecules were retained in the population. All reported results, except for example histograms, show the mean over six repeated runs, with the standard deviation used to calculate error bars. Example histograms show the distribution of metric values for a single run.

For mask generation, we considered the following different values for the mutation rate (i.e., probability that a given token will be masked): [0.15, 0.30, 0.45, 0.60, 0.75]. In addition, three different types of mutation (replacement, insertion, and deletion) were used. For each type, the number of mutations was determined using the binomial distribution for the appropriate number of tokens and mutation rate. A minimum number of 1 mask per molecule was enforced. The locations for each mutation within the molecule string were then randomly sampled. For replacement, the sampled token locations were replaced with a mask. For insertion, one sampled location was used to insert a mask before the given token. Similarly, for deletion, one sampled location was used to delete the token following the mask. The remaining sampled locations for both insertion and deletion were used for replacement.

Fitness in the genetic algorithm simulations was determined using the harmonic mean of multiple molecular metrics. For example, for two metrics ($x_1$ and $x_2$), we used a fitness *F* given by:

1 $$\begin{aligned} F(x_1, x_2) = \frac{2x_1x_1}{x_1+x_2} \end{aligned}$$

By default, we used quantitative estimations of drug-likeness and normalized synthesizability, similar to several previous studies on molecular optimization [15, 16, 41, 42]. To apply the genetic algorithm strategies on a more realistic drug discovery scenario, we also utilized a recently released model for protein binding affinity prediction to generate a molecular metric [43]. Specifically, we used a predicted affinity score for the main protease of SARS-CoV-2. The resulting fitness was, therefore, the harmonic mean of drug-likeness, synthesizability, and the predicted affinity score.

### Molecule data

Similar to previous work [2], we generated a molecule dataset starting from the Enamine *REAL* database [10]. Using a data augmentation strategy with a previously trained language model, we increased the number of molecules to approximately $3.6\cdot 10^{10}$. The strategy for data augmentation is inspired by the pre-training process of the masked language models [2]. The pre-trained models are capable of designing novel, valid and unique molecules by structural rearrangements including combining two molecules. But in order to be selected to augmented data the newly predicted molecules also should be valid, unique and with synthesizability score to be more than certain threshold (in the case 0.30). In preparation for model training, the dataset was partitioned into $7.2\cdot 10^4$ files, each with $5\cdot 10^5$ molecules, stored using the WebDataset [44] library for shared data loading during model training.

In addition to the constructed molecule dataset, we used two additional datasets as the starting population for genetic algorithm simulations. First, we used a subset of $10^5$ molecules from QM9 [45, 46], referred to in the text and figures as GDB9. Second, we selected the top $10^5$ in terms of drug-likeness and synthesizability from a hold-out set of the training data, referred to in the text and figures as Top. These two datasets were used to show the difference in performance for the fixed and adaptive strategies when starting from a relatively low and high initial fitness respectively.

### Language model training

Language model pre-training consists of two different stages, tokenization and mask prediction. During tokenization, a vocabulary is generated for the model based on commonly occurring subsequences within the SMILES string for molecules. Here, we split the SMILES string during pre-processing based on punctuation, which is the default splitting used for the BERT WordPiece tokenizer in the Hugging Face transformers library [47]. The vocabulary for the WordPiece tokenizer was then generated using the full 36 billion molecule dataset, with the vocabulary size set to 32,768.

For mask prediction, we used PyTorch and Hugging Face transformers along with DeepSpeed for distributed training [48]. The transformer architecture that has been used here for the molecule language model is BERT-based. This has approximately 109 million parameters that are learnable. We Pre-train the model with data parallelism technique where each of the GPUs is trained with the model on separate data. As described in [2], we used data parallelism with DeepSpeed’s fused LAMB optimizer to train at scale on a dataset of 3 billion molecules (i.e., the first 6000 partitions of the full molecule dataset). Pre-training was performed on the Summit supercomputer using 1000 nodes (6 Nvidia 16 GB V100 GPUs per node), with each partition of the dataset assigned to a single GPU. We used a batch size of 80 molecules with 3 gradient accumulation steps per GPU, leading to a global batch size of 1.44 million. As stated the primary objective has been to develop a novel algorithm that adapts to initial highly optimized dataset generating similar optimized molecules and not to attain generic distribution of novel molecules or to predict individual molecules with some specific properties. For this purpose, we required a dataset that will be as large as possible to begin with so that the pre-trained model will benefit from learning through the largest chemical dataset available. More so because having trained on as wide a distribution of training data as practicable, we minimize the bias related to data being in or out of distribution in the results of the adaptivity experiment. To have a model that is trained on this large and with wide distribution of molecule dataset we used required large number of GPUs. But once these models are trained, these pre-trained models can be used with one GPU on small dataset for fine-tuning or downstream tasks as required. Pre-training was done for 7 epochs, taking approximately 2.5 h, and model validation was done using mask prediction on a hold-out set of molecules. The best validation accuracy occurred for the final epoch, and the resulting model weights were frozen for language model mutations in the fixed strategy. The model weights were used as the initial conditions for continued training in the adaptive strategy.

**Table 1:** Valid and novel molecules generated by the language model

Mutation rate	0.15	0.30	0.45	0.60	0.75
% valid: GDB9	28	26	21	16	9.7
% novel: GDB9	25	24	20	15	9.2
% valid: TOP	29	31	26	15	4.7
% novel: TOP	26	29	25	15	4.7

To further validate the pre-trained model, we randomly sampled 100,000 molecules with different mutation rates for each of the two data sets used throughout the manuscript as initial populations. New molecules were generated by sampling masked tokens using the Gumbel-softmax layer implemented in PyTorch. We computed the percent of novel and valid molecules present in each population, showing that increasing the mutation rate decreases the number of valid and novel molecules (Table 1).

### Surrogate model for binding affinity

In addition to the heuristic metrics for drug molecules, synthesizability and drug-likeness, we also used an ML model to predict protein binding affinity for a given target, in this case the main protease of SARS-CoV-2. As described in previous work [2], the binding affinity model was generated by fine-tuning language models for both molecule and protein sequences. The output of the model is the predicted negative log (base 10) of the binding affinity. To convert to an affinity score for fitness, we divided the prediction by 10 and clipped the resulting values between 0 and 1. Although the validation and discussion of this model are beyond the scope of the current work, we chose it as an example to illustrate that our proposed optimization strategies can be applied to find high-scoring candidates for both heuristic and ML surrogate scoring models.

## Results

### Fixed and adaptive strategies for molecule generation

Before analyzing the impact of continued language model training on molecule optimization, we considered a simpler task: generating mutations for a fixed set of initial molecules. We implemented this task by using the genetic algorithm without selection (i.e. the parent population remains unchanged). During each generation, mutations are generated and the resulting unique molecules are saved for further analysis. For the fixed strategy, mutations are generated from the fixed pre-trained model, while for the adaptive strategy, the language model is trained for 1 epoch on the initial data in each generation before producing mutations.

**Fig. 2:**
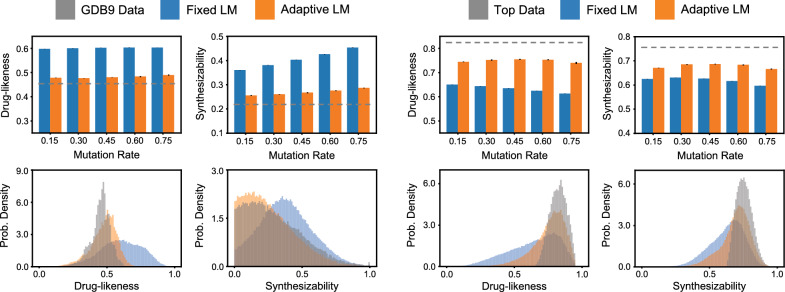
Distributions of molecules produced by a fixed and adaptive approach. Two datasets (GDB9 and a custom dataset with the top scoring molecules for drug-likeness and synthesizability) are used as training data. The fixed approach (blue) generates a broad distribution of molecule scores, while the adaptive approach (orange) more closely mimics the training dataset. Notice that for initial training data with low scores (i.e., GDB9), the adaptive approach produces lower scores on average than the fixed approach, while the situation is reversed for initial training data with high scores (i.e., Top)

As shown in Fig. 2, we used two different initial datasets, GDB9 [46] and Top (see Methods Section - Molecule Data). The mutation rate determines the fraction of tokens that are randomly masked during the generation of new molecules. Each genetic algorithm simulation was run for 5 generations. For each run, the mean drug-likeness and synthesizability scores were calculated for all unique molecules produced in each generation outside of the original data. In terms of time there is no significant difference in generating the molecules between these approaches. For example, the fixed strategy is able to generate $\sim$308k valid molecules in $\sim$42 min out of which $\sim$284k are novel molecules using one GPU while adaptive strategy is able to generate $\sim$250k valid molecules out of which $\sim$212k molecules are novel molecules in $\sim$32 min. The histograms show an example of the distributions for novel molecules with a metric value greater than zero produced from the final generation of a single run with a mutation rate of 0.3.

Due to the continued training of the language model, the mutations generated by the adaptive strategy are much closer, in terms of synthesizability and drug-likeness, to the initial population of molecules. This leads to a decrease in typical values for the GDB9 dataset. However, for the Top molecules, the adaptive strategy produces higher scores. This result can be intuitively explained, as the fixed model is biased by the data used in pre-training (i.e., the pre-trained model will tend to produce mutations that were prevalent in its training dataset). Continued training allows the model to adapt to the new data, either GDB9 or Top.

### Fixed and adaptive strategies for molecule optimization

**Fig. 3:**
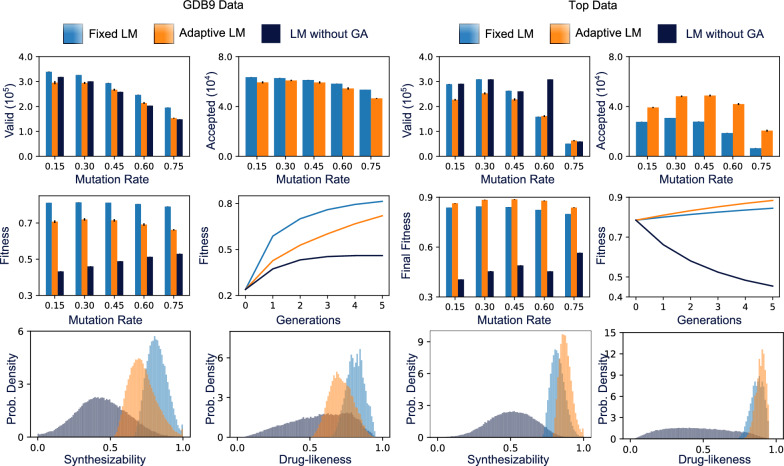
Optimization of molecules for drug-likeness and synthesizability produced by a fixed language model, adaptive language model, or fixed language model without a genetic algorithm based optimization scheme. Two datasets (GDB9 and a custom dataset with the top scoring molecules for drug-likeness and synthesizability) are used as initial data. In the Fitness vs Generations subplots, the y-axis is the average fitness of the population over six runs. The related standard deviations are small compared to the mean values in the order of 0.1%$-$0.2%. The fixed approach (blue) results in a faster increase in fitness, along with greater valid and accepted molecules for the GDB9 dataset. For the top dataset, however, the adaptive approach leads to a faster increase in fitness along with greater accepted molecules. Both the adaptive and fixed approaches outperform the baseline of a fixed language model without the genetic algorithm. The histograms show synthesizability and drug-likeness of the final population after six generations for each approach

For molecular optimization, the ability to adapt to a given initial dataset may or may not be beneficial. In the case of initial data with relatively low scores, we expect the adaptive strategy to slow down optimization, as the generated molecules with have scores similar to the poor initial data. To test this hypothesis, we applied a genetic algorithm (GA) to optimize molecules for drug-likeness and synthesizability starting from the GDB9 dataset. As shown in Fig. 3, the adaptive strategy indeed results in decreased fitness relative to the fixed strategy. This molecular optimization task can be contrasted with the fixed strategy for molecular generation in Fig. 2 as a baseline (shown in dark blue throughout Fig. 3). The fitness plot shown over five generations and histograms of the final molecular populations were generated with a mutation rate of 0.3. Furthermore, the adaptive strategy produced less valid molecules and less accepted molecules (i.e., molecules accepted into the population during selection) for all mutation rates.

The same genetic algorithm applied to the Top dataset produces the opposite results in terms of fitness. Here, the adaptive strategy outperforms the fixed strategy for all mutation rates considered. Interestingly, although the adaptive strategy produces fewer valid molecules for most mutation rates (similar to the GDB9 dataset), it produces more accepted molecules in all cases. The decrease in valid molecules can be understood as adaptive training leading to possible issues with over-fitting the current dataset, rather than learning from the large compound library used for pre-training. However, the increase in accepted molecules suggests that molecular rearrangements learned from a high scoring dataset can improve fitness optimization despite the decrease in valid molecules. The fixed and adaptive GA-based approaches provided much higher fitness than random search despite generating a similar number of valid molecules. For the following analysis, we fixed the mutation rate to 0.3 and focused on ways to use the fixed and adaptive strategies together for molecular design.

### Combining fixed and adaptive strategies

The trade-off in performance for the fixed and adaptive strategies, depending on the distribution of values in the initial dataset, suggests that mixing fixed and adaptive strategies may be useful for molecular optimization. For a new optimization task, a previously optimized dataset will likely not exist to serve as an initial population. In many cases, generating a reasonably optimized dataset may be the entire goal of applying the optimization procedure. Therefore, we assume that the case with poorly optimized initial data, similar to GDB9, is more representative of a typical molecular design problem. In this case, our results have shown that the fixed strategy outperforms the adaptive strategy for optimization. However, as the fitness of the population increases, we expect that the adaptive strategy may provide a better alternative to optimize fitness.

**Fig. 4:**
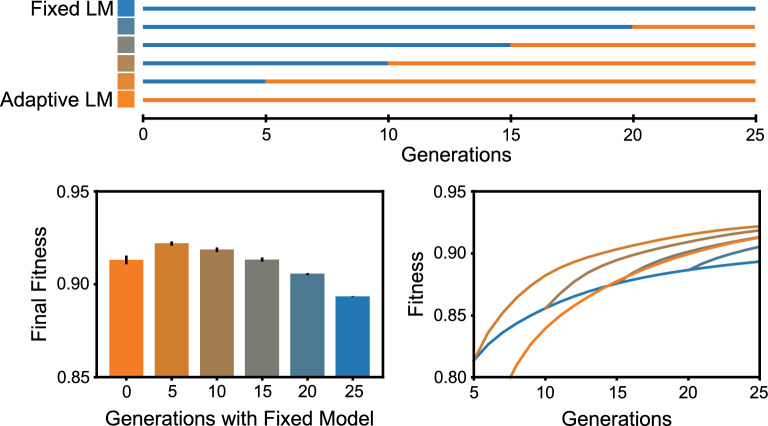
Combining fixed and adaptive approaches during optimization. The fixed approach is used during optimization for 25 epochs. For comparison, the adaptive approach is used starting from the output population of the fixed approach at different generations. The highest fitness is achieved in the case where the adaptive approach is used after 5 epochs of the fixed approach. In the Fitness vs Generations subplots, Y-axis is average fitness of the and calculated as mean over six runs with standard deviations in the order of 0.1–0.2% of mean value. Notice that the adaptive approach starting from the same initial data as the fixed approach achieves a higher fitness after approximately 15 epochs

To test this hypothesis, we implemented various schedules for combining the fixed and adaptive strategies. As show in Fig. 4, the fixed strategy was used initially and then replaced by the adaptive strategy after a specified number of generations. As expected, the optimal strategy involves a combination of the two strategies, with five generations of fixed followed by 20 generations of adaptive. Interestingly, although the purely adaptive strategy (orange) increases much more slowly than the purely fixed strategy (blue), adaptive overtakes fixed in terms of fitness after approximately 15 generations. This suggests that the difficulties associated with fitting more closely to a poor initial dataset can be overcome with the ability to adapt to the population as fitness increases.

### Molecular optimization using a surrogate model

All of the results we have shown so far have used heuristic functions to score molecules (i.e., synthesizability and drug-likeness scores). However, molecular optimization applications may involve additional ML-based surrogate models for scoring. For example, a ML model may be trained on a limited experimental dataset in order to search for molecules with related properties. Here, we use a previously trained surrogate model, which is available for download [43], developed to predict binding affinity for a given protein and molecule. We fix the protein sequence to the main protease of SARS-CoV-2, as described previously [2], and generate a normalized affinity score to use in fitness calculations. During the evaluation of surrogate model for predicting binding affinity for a given protein and molecule, that is also then used in calculating fitness function, the cost of computation increased because the large population size. Due to long runtimes with the surrogate model, a given genetic algorithm simulation was split into multiple sequential jobs, with each job running for five generations. Upon restarting, the model weights were initialized to the fixed pre-trained model.

**Fig. 5:**
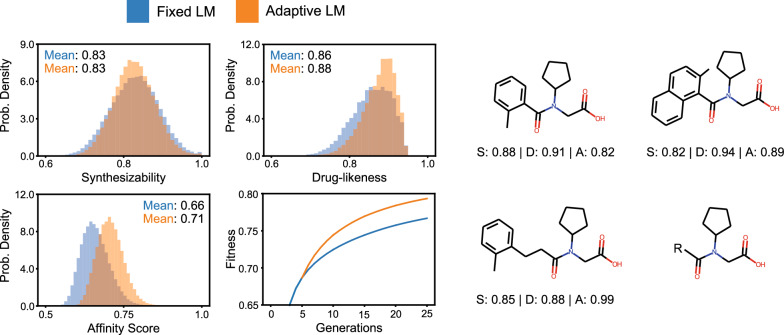
Fixed and adaptive approaches to optimize fitness given by the harmonic mean of synthesizability, drug-likeness, and affinity score. Changing to the adaptive approach after 5 generations results in an increase in fitness as shown by the histograms for drug-likeness and affinity score. The histograms were generated from the final population for the runs with the highest fitness for fixed and adaptive approaches. In the Fitness vs Generations subplots, Y-axis denotes average fitness of the population and plotted as mean over six runs. The related standard deviations are in the order of 0.1%$-$0.2% of the mean values. Sample molecules with similar chemical structures are shown for the adaptive approach. Mutations proposed by the language model show how modifications result in changes in the metrics used to calculate fitness

Building off the results for optimization with heuristic metrics, we compare two optimization schedules. We first apply the fixed strategy for five generations. This is followed by the adaptive strategy for 20 generations, with the continued fixed strategy for comparison. As shown in Fig. 5, the adaptive strategy results in a substantial increase in fitness over the fixed strategy for optimization with the surrogate model. By comparing the histograms for synthesizability, drug-likeness, and affinity score, we determined that the increase in fitness values was primarily the result of increases to the affinity score, suggesting that the adaptive strategy is particularly useful for optimizing the ML scoring model. We also show examples of molecules with different values for the three metrics used during fitness optimization. Beyond generating molecules with high values for all three metrics, the examples show how changes in the chemical structure for a family of molecules result in trade-offs amongst synthesizability, drug-likeness, and affinity score.

## Discussion

### Sequence-only models for drug design

The models presented in this work for both molecule generation and scoring rely only on the molecular sequence (i.e., the SMILES is the only model input). A sequence-only approach is in contrast to ML models that utilized many local and global features (e.g. molecular fingerprints) [3]. Simulation and modeling approaches outside of ML, such as molecular dynamics and docking, use the full three-dimensional structures of both the protein and molecule to predict binding affinity [5]. The primary strength and weakness, therefore, of sequence-only models is the simplicity of the model input. By using SMILES, the model has no direct information concerning geometry or chemical properties. However, SMILES enables the model to be used to train and make predictions on data without known three dimensional structures or previously calculated chemical properties, enabling searches and screening over large portions of chemical space. Furthermore, sequence-only models have been shown to compare favorably to more traditional approaches with manually defined features [49–51].

### Molecule generation through mutations

In this work we have considered molecule generation for design using a language model to generate mutations. This strategy differs from other approaches to develop generative models, such as variational autoencoders [14, 52] and generative adversarial networks [15, 16]. The mutation strategy is dependent on an original molecule in which certain subsequences are changed rather than generating an entire molecule by sampling from a latent space or noise distribution. Although mutation relies upon an original molecule, and thus limits the amount of chemical space accessible for a given round of molecule generation, it has multiple benefits. First, mode collapse should not in principle present a problem for molecule generation through mutation. Because mutations are sampled from each molecule in the population, the full training set is represented in each generation of generated molecules. Second, each round of mutations can be manually inspected along with the scores for each respective molecule, enabling a user to better understand the types of mutations being generated and their impact on fitness. Furthermore, through multiple iterations of mutations and selection, large regions of chemical space can be explored [18], even though a single iteration remains close to the original data.

### Adaptive strategy

As mentioned earlier the masked language models enable us to attain two major design targets - (1) discovering useful molecular representation through tokenization and (2) injecting mutation through masking. In this work our primary objectives are - firstly, given a highly optimized dataset available for initial dataset whether we could devise a certain novel way to generate newer dataset that also guarantees to be optimized in similar fashion. Towards that goal we develop an effective algorithm that overcomes the challenges to generate new set of molecules from an optimized dataset through necessary molecular reconstruction for performing particular different optimization tasks while simultaneously restricting from attaining a generic broader population distribution. In other words, we intend to demonstrate a novel way that will get rid of the certain biases that obstructs necessary structural rearrangements during mutation process, automatically adapt the required chemical region from the original population required for specific user-defined new tasks so that a certain population distribution can be produced. Secondly the other goal of the work is to generate a new population of molecules that are more similar in nature to the original highly optimized data than finding few individual novel molecules with certain specific properties. Together fulfilling both of the above objectives means the improvement that our new strategy offers will be to adapt to specific highly optimized datasets for generating novel molecules that are able to perform highly optimized tasks and be prevented from a broader generic distribution.

## Conclusions

Masked language models coupled with genetic algorithms provide a useful framework for molecular optimization. During tokenization and pre-training, the model determines commonly occurring chemical sequences and rearrangements that can be leveraged for molecule generation through mutations. Furthermore, the language model can be refined using continued training on populations of molecules selected for desired properties. Here, we have shown that the continued training of a language model during genetic algorithm optimization provides a powerful approach to search for molecules according to both heuristic and ML model scoring functions. Models pre-trained on large compounds libraries serve as a useful starting point for both initial optimization from a poorly optimized dataset and initial weights for continued training.

## Data Availability

The source code for this work can be found at https://code.ornl.gov/candle/mlmol in the adaptive-lm directory.

## References

[1] Dong E, Du H, Gardner L (2020). An interactive web-based dashboard to track COVID-19 in real time. Lancet Infect Dis.

[2] Blanchard AE, Gounley J, Bhowmik D, Chandra Shekar M, Lyngaas I, Gao S, Yin J, Tsaris A, Wang F, Glaser J (2022). Language models for the prediction of SARS-CoV-2 inhibitors. Int J High Perform Comput Appl.

[3] Minnich AJ, McLoughlin K, Tse M, Deng J, Weber A, Murad N, Madej BD, Ramsundar B, Rush T, Calad-Thomson S, Brase J, Allen JE (2020). AMPL: a data-driven modeling pipeline for drug discovery. J Chem Inform Model.

[4] Chen H, Engkvist O, Wang Y, Olivecrona M, Blaschke T (2018). The rise of deep learning in drug discovery. Drug Discov Today.

[5] Acharya A, Agarwal R, Baker MB, Baudry J, Bhowmik D, Boehm S, Byler KG, Chen SY, Coates L, Cooper CJ, Demerdash O, Daidone I, Eblen JD, Ellingson S, Forli S, Glaser J, Gumbart JC, Gunnels J, Hernandez O, Irle S, Kneller DW, Kovalevsky A, Larkin J, Lawrence TJ, LeGrand S, Liu S-H, Mitchell JC, Park G, Parks JM, Pavlova A, Petridis L, Poole D, Pouchard L, Ramanathan A, Rogers DM, Santos-Martins D, Scheinberg A, Sedova A, Shen Y, Smith JC, Smith MD, Soto C, Tsaris A, Thavappiragasam M, Tillack AF, Vermaas JV, Vuong VQ, Yin J, Yoo S, Zahran M, Zanetti-Polzi L (2020). Supercomputer-based ensemble docking drug discovery pipeline with application to Covid-19. J Chem Inf Model.

[6] Cho E, Rosa M, Anjum R, Mehmood S, Soban M, Mujtaba M, Bux K, Moin ST, Tanweer M, Dantu S, Pandini A, Yin J, Ma H, Ramanathan A, Islam B, Mey ASJS, Bhowmik D, Haider S (2021). Dynamic profiling of $$\beta$$-coronavirus 3cl mpro protease ligand-binding sites. J Chem Inf Model.

[7] Chen SH, Todd Young M, Gounley J, Stanley C, Bhowmik D (2021). How distinct structural flexibility within sars-cov-2 spike protein reveals potential therapeutic targets. IEEE.

[8] Bhowmik D, Gao S, Young MT, Ramanathan A (2018). Deep clustering of protein folding simulations. BMC Bioinf.

[9] Yang X, Wang Y, Byrne R, Schneider G, Yang S (2019). Concepts of artificial intelligence for computer-assisted drug discovery. Chem Rev.

[10] Enamine REAL Database. https://enamine.net/compound-collections/real-compounds/real-database. Accessed: 2020-04-01 through https://virtual-flow.org/

[11] Martins IF, Teixeira AL, Pinheiro L, Falcao AO (2012). A Bayesian approach to in Silico blood-brain barrier penetration modeling. J Chem Inf Model.

[12] Subramanian G, Ramsundar B, Pande V, Denny RA (2016). Computational modeling of $$\beta$$-secretase 1 (BACE-1) inhibitors using ligand based approaches. J Chem Inf Model.

[13] RDKit: Open-source cheminformatics. http://www.rdkit.org

[14] Jacobs SA, Moon T, McLoughlin K, Jones D, Hysom D, Ahn DH, Gyllenhaal J, Watson P, Lightstone FC, Allen JE, Karlin I, Van Essen B (2021). Enabling rapid COVID-19 small molecule drug design through scalable deep learning of generative models. Int J High Perform Comput Appl.

[15] Blanchard AE, Stanley C, Bhowmik D (2021). Using GANs with adaptive training data to search for new molecules. J Cheminform.

[16] De Cao N, Kipf T (2018) MolGAN: An implicit generative model for small molecular graphs. ICML 2018 workshop on Theoretical Foundations and Applications of Deep Generative Models

[17] Eiben AE, Smith JE (2015). Introduction to evolutionary computing.

[18] Virshup AM, Contreras-García J, Wipf P, Yang W, Beratan DN (2013). Stochastic voyages into uncharted chemical space produce a representative library of all possible drug-like compounds. J Am Chem Soc.

[19] Jensen JH (2019). A graph-based genetic algorithm and generative model/Monte Carlo tree search for the exploration of chemical space. Chem Sci.

[20] Brown N, McKay B, Gilardoni F, Gasteiger J (2004). A graph-based genetic algorithm and its application to the multiobjective evolution of median molecules. J Chem Inform Comput Sci.

[21] Brown N, Fiscato M, Segler MHS, Vaucher AC (2019). GuacaMol: benchmarking models for de novo molecular design. J Chem Inform Model.

[22] Lameijer EW, Kok JN, Bäck T, Ijzerman AP (2006). The molecule evoluator. An interactive evolutionary algorithm for the design of drug-like molecules. J Chem Inform Model.

[23] Nicolaou CA, Apostolakis J, Pattichis CS (2009). De novo drug design using multiobjective evolutionary graphs. J Chem Inform Model.

[24] Lameijer EW, Kok JN, Back T, Ijzerman AP (2006). Mining a chemical database for fragment co-occurrence: discovery of “chemical clichés”. J Chem Inform Model.

[25] Schneider G, Lee ML, Stahl M, Schneider P (2000). De novo design of molecular architectures by evolutionary assembly of drug-derived building blocks. J Comput Aided Mol Design.

[26] Segler MHS, Kogej T, Tyrchan C, Waller MP (2018). Generating focused molecule libraries for drug discovery with recurrent neural networks. ACS Central Sci.

[27] Arés-Pous J, Johansson SV, Prykhodko O, Bjerrum EJ, Tyrchan C, Reymond J-L, Reymond J-L, Chen H, Engkvist O (2019). Randomized smiles strings improve the quality of molecular generative models. J Cheminform.

[28] Flam-Shepherd D, Zhu K, Aspuru-Guzik A (2022). Language models can learn complex molecular distributions. Nat Commun.

[29] Awale M, Sirockin F, Stiefl N, Reymond J-L (2019). Drug analogs from fragment-based long short-term memory generative neural networks. J Chem Inform Model.

[30] Zheng S, Yan X, Gu Q, Yang Y, Du Y, Lu Y, Xu J (2019). Qbmg: quasi-biogenic molecule generator with deep recurrent neural network. J Cheminform.

[31] Méndez-Lucio O, Baillif B, Clevert D-A, Rouquié D, Wichard JD (2018). De novo generation of hit-like molecules from gene expression signatures using artificial intelligence. Nat Commun.

[32] Fabian B, Edlich T, Gaspar H, Segler MHS, Meyers J, Fiscato M, Ahmed M (2020) Molecular representation learning with language models and domain-relevant auxiliary tasks. ArXiv **abs/2011.13230**

[33] Kim H, Na J, Lee WB (2021). Generative chemical transformer: Neural machine learning of molecular geometric structures from chemical language via attention. J Chem Inf Model.

[34] Bagal V, Aggarwal R, Vinod PK, Priyakumar UD (2022). Molgpt: molecular generation using a transformer-decoder model. J Chem Inf Model.

[35] Polykovskiy D, Zhebrak A, Sanchez-Lengeling B, Golovanov S, Tatanov O, Belyaev S, Kurbanov R, Artamonov A, Aladinskiy V, Veselov M, Kadurin A, Johansson S, Chen H, Nikolenko S, Aspuru-Guzik A, Zhavoronkov A (2020). Molecular sets (moses): a benchmarking platform for molecular generation models. Front Pharmacol.

[36] Devlin J, Chang MW, Lee K, Toutanova K (2019) BERT: Pre-training of deep bidirectional transformers for language understanding. NAACL HLT 2019 - 2019 Conference of the North American Chapter of the Association for Computational Linguistics: Human Language Technologies - Proceedings of the Conference 1(Mlm), 4171–4186. arXiv:1810.04805

[37] Blanchard AE, Chandra Shekar M, Gao S, Gounley J, Lyngaas I, Glaser J, Bhowmik D (2022). Automating genetic algorithm mutations for molecules using a masked language model. IEEE Trans Evolut Comput.

[38] Weininger D (1998). SMILES, a chemical language and information system. 1. Introduction to methodology and encoding rules. J Chem Inf Comput Sci.

[39] Schuster M, Nakajima K (2012) Japanese and korean voice search. In: 2012 IEEE International Conference on Acoustics, Speech and Signal Processing (ICASSP), pp. 5149–5152. 10.1109/ICASSP.2012.6289079

[40] Wu Y, Schuster M, Chen Z, Le QV, Norouzi M, Macherey W, Krikun M, Cao Y, Gao Q, Macherey K, Klingner J, Shah A, Johnson M, Liu X, Kaiser Ł, Gouws S, Kato Y, Kudo T, Kazawa H, Stevens K, Kurian G, Patil N, Wang W, Young C, Smith J, Riesa J, Rudnick A, Vinyals O, Corrado G, Hughes M, Dean J (2016) Google’s Neural Machine Translation System: Bridging the Gap between Human and Machine Translation, 1–23. arXiv:1609.08144

[41] Bickerton GR, Paolini GV, Besnard J, Muresan S, Hopkins AL (2012). Quantifying the chemical beauty of drugs. Nat Chem.

[42] Ertl P, Schuffenhauer A (2009). Estimation of synthetic accessibility score of drug-like molecules based on molecular complexity and fragment contributions. J Cheminf.

[43] jglaser/protein-ligand-mlp-1. https://huggingface.co/jglaser/protein-ligand-mlp-1

[44] Aizman A, Maltby G, Breuel T (2019) High performance I/O for large scale deep learning. In: 2019 IEEE International Conference on Big Data (Big Data), pp. 5965–5967. IEEE

[45] Ramakrishnan R, Dral PO, Rupp M, Von Lilienfeld OA (2014). Quantum chemistry structures and properties of 134 kilo molecules. Sci Data.

[46] gdb9 Dataset. http://deepchem.io.s3-website-us-west-1.amazonaws.com/datasets/gdb9.tar.gz. Accessed 28 May 2021

[47] Wolf T, Debut L, Sanh V, Chaumond J, Delangue C, Moi A, Cistac P, Rault T, Louf R, Funtowicz M, Davison J, Shleifer S, von Platen P, Ma C, Jernite Y, Plu J, Xu C, Scao TL, Gugger S, Drame M, Lhoest Q, Rush AM (2020) Transformers: State-of-the-art natural language processing. In: Proceedings of the 2020 Conference on Empirical Methods in Natural Language Processing: System Demonstrations, pp. 38–45. Association for Computational Linguistics, Online. https://www.aclweb.org/anthology/2020.emnlp-demos.6

[48] Rajbhandari S, Rasley J, Ruwase O, He Y (2020) Zero: Memory optimizations toward training trillion parameter models. International Conference for High Performance Computing, Networking, Storage and Analysis, SC 2020-Novem, 1–24. 10.1109/SC41405.2020.00024.arXiv:1910.02054

[49] Wang S, Guo Y, Wang Y, Sun H, Huang J (2019) Smiles-Bert: Large scale unsupervised pre-training for molecular property prediction. ACM-BCB 2019 - Proceedings of the 10th ACM International Conference on Bioinformatics, Computational Biology and Health Informatics, 429–436. 10.1145/3307339.3342186

[50] Xue D, Zhang H, Xiao D, Gong Y, Chuai G, Sun Y, Tian H, Wu H, Li Y, Liu Q (2020). X-MOL: large-scale pre-training for molecular understanding and diverse molecular analysis. bioRxiv.

[51] Kim H, Lee J, Ahn S, Lee JR (2021). A merged molecular representation learning for molecular properties prediction with a web-based service. Sci Rep.

[52] Gómez-Bombarelli R, Wei JN, Duvenaud D, Hernández-Lobato JM, ánchez-Lengeling B, Sheberla D, Aguilera-Iparraguirre J, Hirzel TD, Adams RP, Aspuru-Guzik A, (2018) Automatic chemical design using a data-driven continuous representation of molecules. ACS Central Sci 4(2):268–276 10.1021/acscentsci.7b00572 PMC5833007 29532027

